# Supplementary material for: Regulatory context drives conservation of glycine riboswitch aptamers
Source: PLoS Comput Biol. 2019 Dec 20;15(12):e1007564. doi: 10.1371/journal.pcbi.1007564 (PMC6944388; doi:10.1371/journal.pcbi.1007564)
Supplement: Attachment — Submitted filename: Review_PLoS-CompBio_2019.pdf [file pcbi.1007564.s033.pdf]

Review Report

The manuscript addresses an important problem related to evolution of glycine riboswitches by tracing the evolutionary pathway that relates singlet glycine riboswitches to their tandem counterparts. In the process it resolves an existing conundrum pertaining to the relative importance of the two aptamers on regulation of the downstream gene. The paper also highlights the important role of genomic context on the evolution of glycine riboswitches, an aspect that has been pointed out in the literature, for riboswitches belonging to few other classes.

The manuscript is well-written, the analysis novel and rigorous and the conclusions well supported by the evidence provided. The graph clustering method is the most interesting aspect of the analysis since it validates as well as supplements the results of the phylogenetic analysis.

I have suggestions for a few minor revisions following which the manuscript can be accepted for publication.

Line 255-256 should read: “We first categorized singleton aptamers within our 254 dataset (includes all bacteria, refseq77-microbial) into singleton type-1 or singleton type-2 based on whether the ghost aptamer was found 3’ or 5’ of the glycine aptamer.” To make it consistent with the definition of singletons given in lines 137-138.

Fig.3 & 5: In part B, the colour-coding of the nodes is not clearly explained. I suppose the background colour indicates a specific community? The communities are quite clear from part A itself and the community detection using R as depicted in part B may be useful in reinforcing the evidence already seen in part A if the colour of the nodes is made consistent with those in part A.

Fig. 7: It will be useful to label the Type 1 and Type 2 singletons in Fig. 7

Line 377: Horizontal transfer of riboswitches may be difficult to detect, but indirect evidence of such horizontal transfer can come from evidence of horizontal transfer of the riboswitch-regulated gene. The authors should check if there is any such evidence of horizontal transfer of TP and GCV genes by constructing individual gene trees. Clustering of one or more species belonging to one family with those belonging to another family in the gene trees would suggest the plausibility of horizontal transfer of riboswitches along with the regulated gene.

Lines 445-451: From the description it was not very clear to me whether the covariance model built from both aptamers of tandem riboswitches were used to identify singletons as well or if two separate covariance models based on one of the two aptamers of a tandem riboswitch were used to detect singletons, in addition to a tandem covariance model used to detect tandems.

Line 477: should read “…higher similarity *than* the threshold…”

Important riboswitch-specific references are missing. The authors need to expand their reference list and not ignore contributions of other groups to riboswitch discovery and mapping of their genomic context. Relevant references are

Abreu-Goodger C, Merino E (2005) RibEx: a web server for locating riboswitches and other conserved bacterial regulatory elements. *Nucleic Acids Res* 33: W690±W692.

Chang T-H, Huang H-D, Wu L-C, Yeh C-T, Liu B-J, Horng J-T (2009) Computational identification of riboswitches based on RNA conserved functional sequences and conformations. *RNA:*15(7):1426-1430.

Havill, J.T., et al. (2014) A new approach for detecting riboswitches in DNA sequences. *Bioinformatics*, 30, 3012–3019

Mukherjee S, Sengupta S (2016) Riboswitch Scanner: an efficient pHMM-based web-server to detect riboswitches in genomic sequences. *Bioinformatics* 32(5):776±778.

Mukherjee, S., et al. (2019) RiboD: a comprehensive database for prokaryotic riboswitches. *Bioinformatics*, 35, 3514-3543.
