# Supplementary material for: Regulatory context drives conservation of glycine riboswitch aptamers
Source: PLoS Comput Biol. 2019 Dec 20;15(12):e1007564. doi: 10.1371/journal.pcbi.1007564 (PMC6944388; doi:10.1371/journal.pcbi.1007564)
Supplement: Attachment — Submitted filename: Crum2019_PLoS_Rebuttal.docx [file pcbi.1007564.s034.docx]

Round 1 Reviews:

**Reviewer 1**:

Reviewer #1: Crum, Ram-Mohan and Meyer have explored the evolutionary relationships

between singleton and tandem glycine, using a number of different

approaches. This provides some evidence for which switch (5` or 3`) of

the tandem riboswitches is governing gene expression.

The number of phylogenetically informative sites in short RNAs is

generally very small, hence trees for these are often quite poor. With

doublet evolutionary models [1,2], using INDEL-aware evolutionary

models [3] and flanking sequence [4] being three strategies that have been

employed for addressing this problem.

The authors have used a number of approaches to ensure that their

results are robust, including more traditional methods (e.g. RaxML)

and non-traditional approaches such as RNAMountAlign. I am slightly

worried about the latter, if it's based upon the mountain metric

proposed by Moulten et al (2000), then there is an issue that the

outer-most basepairs in stems have too large an impact on the result,

furthermore the "correction" proposed by Moulton et al, fails to

correct the problem (based upon my own simulations). While this

shouldn't be a problem for this study, I suggesting treating this

method with caution.

We thank the reviewer for their concern and apt observation regarding RNAMountAlign. We are aware of the limitations of exclusively using mountain height as a metric. For this reason we have utilized 4 other tools for comparison (RNApdist, Dynalign, FoldAlign, and Clustal-Omega) which do not utilize mountain height to validate our findings.

MAJOR COMMENTS:

1. extensive use of graph clustering has been used, was bootstrapping or similar employed to ensure the clusters are robust? What about alternative clustering approaches e.g. MCL or DBSCAN?

We thank the reviewer for their thorough and helpful feedback. In regards to alternative clustering approaches, repeating our clustering using alternative clustering metrics results in similar groupings, though not identical.  MCL clustering results in slightly larger clusters, though the core finding of specific aptamer types clustering together does not change (namely: aptamer 1 of tandem and singleton type-1 clustering, as well as aptamer 2 of tandems clustering with singleton type-2). For the *Bacilli* aptamers, we used an RNAmountAlight threshold of 8 instead of 12 (as we normally visualize) because MCL was unable to reach an equilibrium when run on the network using a threshold of 12. This did not significantly affect our findings. DBSCAN was less conclusive, as the widely varying densities of the clusters confounded selection of an ϵ-neighborhood value appropriate for clustering of the network as a whole. Low ϵ values were sufficient for identifying the most highly similar aptamers (ie: aptamers of the same type coming from very closely related bacteria), while higher ϵ values were necessary for patterned clustering of less closely related aptamers, but had a tendency to over-cluster in many cases. For this reason, clustering of groups similar to those identified in the original study was able to be achieved, but it required a range of ϵ-neighborhood values to be accomplish. Due to the high variance in group densities within the network and the noisiness of the data, we do not believe that dbscan is the best algorithm for evaluating our data.

We addressed this within the text by adding a sentence to the methods and including 4 supplemental figures (Supplemental Figures S4, S5, S9, and S10) showing the MCL and DBSCAN clustering:

“Cluster composition was also validated by MCL and DBSCAN clustering as well”

With regards to bootstrapping, we performed 100 replicates of a parametric bootstrapping analysis which perturbs 5% of the network and then re-clusters. This analysis perturbs the network by adding/removing edges at random in a 1:1 ratio, resulting in a network that contains the same nodes and an equivalent number of edges, but 5% of the edges connect different nodes. For each iteration, we determined the new clustering for our group of interest using our community detection methods (include methods here?). These were then compared to the original group that had no perturbation. This comparison was done across all 100 iterations and uses Jaccard Similarity to calculate similarity of each post-perturbation cluster to the original cluster. Average Jaccard Similarity across the 100 iterations was used to determine robustness for our clusters. We found that while three of our four community detection algorithms used provided reasonable confidence in cluster stability (particularly cluster_walktrap which had consistently very high Jaccard Similarity after bootstrapping), cluster_fast_greedy did not provide high Jaccard Similarities. We believe this is because the algorithm seems to be more sensitive to over-grouping distinct nodes/clusters into one group based on relatively low connectivity. When bootstrapping, this results in our cluster of interest remaining in the same group, but that group being bundled in with other groups that the other algorithms recognized as distinct. Despite cluster_fast_greedy’s low Jaccard Similarity, the overage Jaccard Similarity across the other three metrics gives us confidence in the stability of our identified networks.

We have added an explanation of this to the methods, as well as added a supplemental figure (Supplemental Figure S3) to show Jaccard Similarity Indexes after bootstrapping.

“Following cluster identification, we performed 100 replicates of a parametric bootstrapping analysis which perturbs 5% of the network and then re-clusters. This analysis perturbs the network by adding/removing edges at random in a 1:1 ratio, resulting in a network that contains the same nodes and an equivalent number of edges, but 5% of the edges connect different nodes. For each iteration, we determined the new clustering for our group of interest using the igraph community detection methods. These were then compared to the original group that had no perturbation. This comparison was done across all 100 iterations and uses Jaccard Similarity Index to calculate similarity of each post-perturbation cluster to the original cluster. Average Jaccard Similarity Index across the 100 iterations was used to determine robustness for our clusters.”

Text was added to both the *Bacilli* and *Actinobacteria* section of the results to inform the reader of cluster stability validation:

“Cluster stability was verified using 100 replicates of parametric bootstrapping (**Supplementary Figure S3**) (see methods), as well as comparison to MCL (**Supplementary Figure S4**) and DBSCAN **Supplementary Figure S5**) clustering output.”

2. If the tandem alignment/CM had been split into 5` and 3` halves, then the two half CMs could have also been used to determine which half the singletons are likely derived from.

We recognize the potential for the bifurcation of a tandem covariance model into two singleton models to classify singletons. While it may have been possible to classify some singletons using a split tandem CM, singleton CMs which do not include a ghost aptamer and kink-turn have not performed well in singleton type classification. This is most easily demonstrated with the RFAM singleton CM, which picks up tandem aptamer 1 and tander apamer 2, as well as aptamers belonging to both Type I and Type II singletons. Splitting our tandem CM  would likely result in two CMs that perform similarly to the RFAM CM, as both the RFAM and our tandem CM are generated using a range of riboswitches regulating multiple genomic contexts. Adding the ghost aptamer and kink-turn is more likely to improve the ability of Infernal to distinguish these aptamer types.

If we generated two tandem CMs, one from riboswitches regulating GCV and another from riboswitches regulating TP, we might be able to improve the specificity of the split CMs by using the first aptamer of the GCV tandem CM and the second aptamer of the TP tandem CM. However, this also *a priori* assumes the result we obtained, and thus leads to circular arguments.

3. It is assumed that singletons are exclusively derived from either a 5` or 3` half of a tandem ancestor, yet half 5` + half 3` hybrids have not been excluded as a possibility.

E.g.

tandem 5555555555555555555lllll33333333333333333333

5p-single 5555555555555555555-------------------------

3p-single ------------------------33333333333333333333

5p3p-hybrid 5555555555------------------------3333333333

We acknowledge the possibility that a subset of singletons identified may have arisen from half 5` + half 3` hybrids of tandem riboswitches. However, a hybridization of half 5` + half 3` would not account for the presence, and demonstrated necessity, for the ghost aptamer seen in functional singleton glycine riboswitches. While hybrid singletons may exist, they would either be identified in this work as type-0 aptamers, or result from the de novo evolution of the ghost aptamer.  However, most type-0 aptamers in our dataset are unclassified not due to lack of a convincing ghost aptamer, but rather to the presence of two flanking hairpins and inability to conclusively determine which is the best choice in the absence of experimental data.

To clarify for this possibility we added to the discussion:

 “It is possible that some singletons may have arisen from deletion of the middle section of a tandem riboswitch, leaving the 5’ half of aptamer 1 and the 3’ half of aptamer 2, resulting in a singleton. However, this scenario seems unlikely because it does not account for the ghost aptamer, which is important for structural stability of the glycine riboswitch.”

4. I didn't really understand the network density plots, are they flipped cumulative distributions?

We thank the reviewer for bringing this lack of clarity to our attention. The network density plots are indeed equitable to flipped cumulative distributions. While traditional cumulative distribution functions represent the probability of a randomly selected value falling below a threshold, we describe the network density plots as representing the proportion of edges within a cluster which are greater than a given threshold. This is equivalent to describing them as cumulative distributions which describe the probability of a randomly selected value being greater than a given threshold. To clarify this point, we have supplemented the results and methods sections:

Methods: “These generated graphs equate to a flipped cumulative distribution of possible edges and actual edges for a cluster as we threshold the network based on edge weights.”

5. Figures 2, 3 5 and 6 are very similar, are they all necessary?

We thank the reviewer for their assessment, and recognize that the mentioned figures do have repetitive elements. For this reason, we will be moving the entirety of figure 5 and sub-figures 6C/D to the supplemental material.

MINOR COMMENTS:

1. what was the distribution of distances between the 2 halves of tandem models? This would help justify the "100 nucleotides" threshold.

The vast majority of tandem riboswitches which passed our filtering have their first and second aptamer within 25 nucleotides of each other. The farthest distance between two aptamers within a tandem riboswitch is 32 nucleotides. The lack of tandem riboswitches with aptamers farther than 35 nucleotides apart makes us believe that a threshold of 100 nucleotides is reasonable.

We also added a sentence to the methods briefly describing this distribution to the methods. “...riboswitch; the largest distance between tandem aptamers observed was 32 nts”

2. Ralee is often used as well as R2R in order to curate alignments, was this also tried?

Our alignments only underwent minor modifications during curation, and Ralee was used at points throughout the process to visualize conserved structure of the alignments. We have added a sentence to the methods crediting/citing the use of Ralee (and VARNA) in manual curation:

“Ralee was then used to perform minor curation of alignments and VARNA was implemented for secondary structure visualization throughout the process (78, 79).”

3. Were the GA thresholds provided by Rfam not useful, as opposed to an E-value threshold?

The GA thresholds the RFAM provides did offer a starting threshold for our analysis. However, using the GA threshold resulted in ~98.5% of identified sequences passing the curation process to be qualified as a riboswitch. We opted for a more stringent threshold, which resulted in ~71% of sequences passing this curation step, in order to be more confident that the sequences used in our analysis represent true glycine riboswitches.

4. Were expression platforms included or excluded from the CM models?

Expression platforms were not explicitly included within the CMs used. This is because the glycine riboswitches are found regulating a range of different genes using different mechanisms of action, thus their expression platforms vary widely. We have noted this within the methods:

“The generated model is able to identify tandem glycine riboswitch aptamers, but does not explicitly include the expression platform due to the diversity of mechanisms of action for the glycine riboswitch.”

5. Pg. 19, had forgotten what a "ghost" aptamer is by the time I encountered it again.

We thank the reviewer for their insight and have added a few sentences as recap for the ghost aptamer within the discussion to prep the reader before describing our model.

Added reminder to  “Riboswitch Identification” section of Materials and Methods: “an adjacent stem structure that functions as a scaffold for tertiary interaction with the ligand-binding aptamer.”

REFERENCES:

1. Schöniger M, von Haeseler A. Toward assigning helical regions in alignments of ribosomal RNA and testing the appropriateness of evolutionary models. Journal of molecular evolution. 1999 Nov 1;49(5):691-8.

2. Gesell T, Von Haeseler A. In silico sequence evolution with site-specific interactions along phylogenetic trees. Bioinformatics. 2005 Dec 6;22(6):716-22.

3. Rivas E, Eddy SR. Probabilistic phylogenetic inference with insertions and deletions. PLoS computational biology. 2008 Sep 19;4(9):e1000172.

4. Pignatelli M, Vilella AJ, Muffato M, Gordon L, White S, Flicek P, Herrero J. ncRNA orthologies in the vertebrate lineage. Database. 2016 Jan 1;2016.

**Reviewer 2**:

The manuscript addresses an important problem related to evolution of glycine riboswitches by

tracing the evolutionary pathway that relates singlet glycine riboswitches to their tandem

counterparts. In the process it resolves an existing conundrum pertaining to the relative importance of the two aptamers on regulation of the downstream gene. The paper also highlights the important role of genomic context on the evolution of glycine riboswitches, an aspect that has been pointed out in the literature, for riboswitches belonging to few other classes.

The manuscript is well-written, the analysis novel and rigorous and the conclusions well supported by the evidence provided. The graph clustering method is the most interesting aspect of the analysis since it validates as well as supplements the results of the phylogenetic analysis.

I have suggestions for a few minor revisions following which the manuscript can be accepted for

publication.

Line 255-256 should read: “We first categorized singleton aptamers within our 254 dataset (includes all bacteria, refseq77-microbial) into singleton type-1 or singleton type-2 based on whether the ghost aptamer was found 3’ or 5’ of the glycine aptamer.” To make it consistent with the definition of singletons given in lines 137-138.

We thank the reviewer for their attention to detail and the crucial correction. We have made the suggested changes to clarify our text.

Fig.3 & 5: In part B, the colour-coding of the nodes is not clearly explained. I suppose the background colour indicates a specific community? The communities are quite clear from part A itself and the community detection using R as depicted in part B may be useful in reinforcing the evidence already seen in part A if the colour of the nodes is made consistent with those in part A.

We thank the reviewer for their observation. As the node colors and background within the clusters are set and linked by the clustering algorithm functions, decoupling node colors from background bubble colors has proven difficult. For this reason we were unable to set node colors to correspond to aptamer type while maintaining background bubbles corresponding to clusters. We have, however, added text to the legends of these two figures to clarify that node colors correspond to distinct clusters for part B:

“Node colors correspond to distinct clusters detected.”

Fig. 7: It will be useful to label the Type 1 and Type 2 singletons in Fig. 7

We thank the reviewer for their input. We have added the clarifying labels to figure 7.

Line 377: Horizontal transfer of riboswitches may be difficult to detect, but indirect evidence of such horizontal transfer can come from evidence of horizontal transfer of the riboswitch-regulated gene.

The authors should check if there is any such evidence of horizontal transfer of TP and GCV genes by constructing individual gene trees. Clustering of one or more species belonging to one family with those belonging to another family in the gene trees would suggest the plausibility of horizontal transfer of riboswitches along with the regulated gene.

We thank the reviewer for their feedback. We have conducted the proposed analysis and found that there are not enough horizontal gene transfer events implied in the data to account for the observed divergence across genomic contexts. A tree generated using sodium:amino-acid symporters regulated by glycine riboswitches found little-to-no evidence of widespread horizontal gene transfer (Supplementary Figure S16). A similar tree generated using the aminomethyltransferase gene from the glycine cleavage operon found that genes originating from firmicutes clusters closely with a clade from proteobacteria, but is not contained within the clade (Supplementary Figure S15). This could be potential evidence of horizontal gene transfer between proteobacteria and firmicutes.

We have addressed this within the text by adding a few sentences to the discussion and including two supplemental figures showing gene trees for aminomethyltransferase and symporter genes regulated by glycine riboswitches:

“Widespread horizontal transfer of the riboswitch with its accompanying gene could account for our findings. To investigate this possibility, we generated gene trees for aminomethyltransferases and symporters preceded by glycine riboswitches. From these trees, there is limited evidence of horizontal transfer of these genes”

Lines 445-451: From the description it was not very clear to me whether the covariance model built from both aptamers of tandem riboswitches were used to identify singletons as well or if two separate covariance models based on one of the two aptamers of a tandem riboswitch were used to detect singletons, in addition to a tandem covariance model used to detect tandems.

We thank the reviewer for bringing this source of confusion to our attention. We have added to the text to clarify that the RFAM covariance model identifies individual aptamers, which was then used to generate a tandem model based on aptamers within 100nt or each other, and then that model was used to supplement the dataset with any tandems that the RFAM model may have missed:

Infernal’s cmsearch function was used to query all RefSeq77 bacterial genomes using the RFAM glycine riboswitch covariance model to identify **all individua**l putative glycine riboswitch aptamers (RF00504) (13,14,47). ... This tandem covariance model was then used to query the RefSeq77 bacterial database and supplement our current set of putative glycine riboswitches **with any tandems that may have been missed by the RFAM covariance model**.

Line 477: should read “...higher similarity than the threshold...”

We appreciate the reviewers attention to detail and have corrected the mistake within the sentence.

Important riboswitch-specific references are missing. The authors need to expand their reference list and not ignore contributions of other groups to riboswitch discovery and mapping of their genomic context. Relevant references are

Abreu-Goodger C, Merino E (2005) RibEx: a web server for locating riboswitches and other conserved

bacterial regulatory elements. Nucleic Acids Res 33: W690±W692.

Chang T-H, Huang H-D, Wu L-C, Yeh C-T, Liu B-J, Horng J-T (2009) Computational identification of

riboswitches based on RNA conserved functional sequences and conformations. RNA:15(7):1426-

1430.

Havill, J.T., et al. (2014) A new approach for detecting riboswitches in DNA sequences. Bioinformatics,

30, 3012–3019

Mukherjee S, Sengupta S (2016) Riboswitch Scanner: an efficient pHMM-based web-server to detect

riboswitches in genomic sequences. Bioinformatics 32(5):776±778.

Mukherjee, S., et al. (2019) RiboD: a comprehensive database for prokaryotic riboswitches.

Bioinformatics, 35, 3514-3543.

We thank the reviewer for pointing out the gap within our background of our introduction. These papers have been cited in the introduction when discussing other approaches for detection and/or clustering of riboswitches:

“Other approaches for detection and clustering of structured RNAs provide robust methods for researchers to classify members of existing RNA families (12–19) and identify novel structured ncRNA (20–25).”
